# Modeling acute respiratory illness during the 2007 San Diego wildland fires using a coupled emissions-transport system and generalized additive modeling

**DOI:** 10.1186/1476-069X-12-94

**Published:** 2013-11-05

**Authors:** Brian Thelen, Nancy HF French, Benjamin W Koziol, Michael Billmire, Robert Chris Owen, Jeffrey Johnson, Michele Ginsberg, Tatiana Loboda, Shiliang Wu

**Affiliations:** 1 Michigan Tech Research Institute, Michigan Technological University, 3600 Green Court, Suite 100, Ann Arbor, MI 48105, USA; 2 San Diego County Health & Human Services Agency, 3851 Rosecrans Street, San Diego, CA 92110, USA; 3 Department of Geographical Sciences, University of Maryland, 2181 LeFrak Hall, College Park, MD 20742, USA; 4 Department of Geological and Mining Engineering and Sciences and Department of Civil and Environmental Engineering, Michigan Technological University, 1400 Townsend Drive, Houghton, Michigan 49931, USA; 5 Currently at NESII/CIRES/NOAA Earth System Research Laboratory, 325 Broadway, Boulder, CO 80305, USA; 6 Currently at U. S. Environmental Protection Agency, Office of Air Quality Planning and Standards, Research Triangle Park, Durham, NC 27711, USA

**Keywords:** Wildland fire, Particulate matter emissions, Syndromic surveillance, Generalized additive modeling, Air quality, Respiratory health, San Diego County, California

## Abstract

**Background:**

A study of the impacts on respiratory health of the 2007 wildland fires in and around San Diego County, California is presented. This study helps to address the impact of fire emissions on human health by modeling the exposure potential of proximate populations to atmospheric particulate matter (PM) from vegetation fires. Currently, there is no standard methodology to model and forecast the potential respiratory health effects of PM plumes from wildland fires, and in part this is due to a lack of methodology for rigorously relating the two. The contribution in this research specifically targets that absence by modeling explicitly the emission, transmission, and distribution of PM following a wildland fire in both space and time.

**Methods:**

Coupled empirical and deterministic models describing particulate matter (PM) emissions and atmospheric dispersion were linked to spatially explicit syndromic surveillance health data records collected through the San Diego Aberration Detection and Incident Characterization (SDADIC) system using a Generalized Additive Modeling (GAM) statistical approach. Two levels of geographic aggregation were modeled, a county-wide regional level and division of the county into six sub regions. Selected health syndromes within SDADIC from 16 emergency departments within San Diego County relevant for respiratory health were identified for inclusion in the model.

**Results:**

The model captured the variability in emergency department visits due to several factors by including nine ancillary variables in addition to wildfire PM concentration. The model coefficients and nonlinear function plots indicate that at peak fire PM concentrations the odds of a person seeking emergency care is increased by approximately 50% compared to non-fire conditions (40% for the regional case, 70% for a geographically specific case). The sub-regional analyses show that demographic variables also influence respiratory health outcomes from smoke.

**Conclusions:**

The model developed in this study allows a quantitative assessment and prediction of respiratory health outcomes as it relates to the location and timing of wildland fire emissions relevant for application to future wildfire scenarios. An important aspect of the resulting model is its generality thus allowing its ready use for geospatial assessments of respiratory health impacts under possible future wildfire conditions in the San Diego region. The coupled statistical and process-based modeling demonstrates an end-to-end methodology for generating reasonable estimates of wildland fire PM concentrations and health effects at resolutions compatible with syndromic surveillance data.

## Background

In 2007, catastrophic wildland fires burned approximately 650,000 acres of southern Californian grassland and chaparral. Drought-level rainfall, fire-susceptible vegetation fuels, high temperatures, and strong Santa Ana winds created an ideal fire weather environment for fast moving, high intensity burns. Fires of this type are not unusual in the region; coastal southern California is classified as a Mediterranean eco-climatic zone dominated by fire-adapted chaparral shrublands [1]. It has been shown that the largest and longest duration wildfires in southern California are often accompanied by Santa Ana winds [2], a seasonal weather phenomenon affecting southern California from Los Angeles to San Diego that typically occurs from September to April [3,4]. Being weather-driven events, the impacts of a changing climate are of concern for the future. Models of future climate generally predict weather conditions that favor an increase in the occurrence and severity of California wildland fires (i.e. periods of low annual precipitation and high average daily temperatures [5]) and long-term trends conducive to lengthened fire season.

This study helps to address the impact of fire emissions on human health within the context of a changing climate by modeling the exposure potential of proximate populations to atmospheric particulate matter (PM) from vegetation fires. In this study, an empirical model describing PM emissions was coupled with an atmospheric advection and dispersion model to derive daily map-based estimates of PM concentrations resulting from wildfire. Using historic data from the 2007 southern California wildland fires, fire-derived PM concentration was linked to spatially explicit syndromic surveillance data collected through the San Diego Aberration Detection and Incident Characterization (SDADIC) system [6].

The County of San Diego established syndromic surveillance infrastructure starting in 2001 with a goal to monitor the health of the population in order to detect health impacts caused by disease outbreaks, natural disasters, high profile events, or bioterrorist events. The goal of SDADIC was to establish a capacity for the rapid detection of emerging public health events, bioterrorism, monitoring for special events and natural disasters. This system has been used following 9/11, the 2003 Super Bowl, several natural disasters, the H1N1 Pandemic and other high profile events. Syndromic surveillance supplements traditional surveillance sources, such as legally reportable communicable disease reports and death certificate data. SDADIC has been valuable in the detection of valid biological, chemical and radiological events which led to follow-up and in some cases public health interventions. This coupled with the experience in other jurisdictions, lends confidence of the system to detect population impact from a natural disasters. While syndromic surveillance data for the region is available starting in 2003, for the early years (2003 to 2005) only a few hospitals were part of the system, so while 2003 was a high fire year it was not used in this analysis. By 2007, 16 hospital emergency departments were regularly reporting to the system which provided data representing 86% of county emergency department visits for this study.

Previous analysis has shown an impact on health due to decreased air quality during the 2003 catastrophic fire events in San Diego [6,7]. Smoke from vegetation fires is known to be an important contributor to poor air quality and impacts on human health. There is ample evidence of the role of fire in altering air quality and as a source of carbon-based atmospheric compounds [8,9]. Human health effects from elevated PM concentrations are well documented; inhalation of smoke from wildland fire has been linked to respiratory illnesses such as dyspnea, asthma, and chronic obstructive pulmonary disease (COPD) [7,10]. Recent research has focused on linking point source fire emissions to local PM “events” during which people who are in the path of the smoke plume experience PM concentrations far exceeding the average daily concentrations [11,12]. These PM events lead directly to increased reports of acute respiratory illness (i.e. asthma, dyspnea, COPD) in exposed individuals, an effect evidenced by syndromic surveillance data maintained by county public health officials and other health monitoring methods [7,11,13,14].

Wildfire smoke is one of the components tracked within the National Oceangraphic and Atmospheric Administration (NOAA) National Air Quality Forecast system [15], using the US Forest Service BlueSky modeling framework (http://www.airfire.org/bluesky/). BlueSky is an experimental framework which has provided tools for quantifying emissions and assessing air quality conditions modified by wildland fire that is a valuable resource where available. However, currently there is no operational methodology to model and forecast wildfire PM concentrations across regional domains. Operational systems to predict potential respiratory health effects of PM plumes from wildland fires are also not in place, in part due to a lack of methodology for rigorously and systematically relating the two. This paper reviews a study that draws from previous work and provides some alternative approaches to model fire’s impact on respiratory health compared to previous research activities. These previous studies used various methods to map fire emissions concentrations and relate these estimated smoke conditions to health outcomes [7,11,14]. Here we present a statistical approach that is more rigorous than these previous studies, which have used only linear models. The approach presented uses modeled smoke concentrations based on an ecologically-centered method of quantifying fire emissions, a method consistent with the BlueSky Framework, rather than interpoleated air quality monitoring data found in most other studies. It also employs a well-established syndromic surveillance data system that has been proven to have value for epidemiological study and emergency response. The contribution of this paper specifically targets that absence by modeling the emission, transmission, and distribution of PM following a wildland fire in both space and time in order to rigorously connect PM concentration to health outcomes. The coupled statistical and process-based modeling methodology presented in this paper demonstrates an end-to-end method for generating reasonable estimates of wildland fire particulate matter concentrations and health effects at resolutions compatible with resolutions (temporal and spatial) that syndromic surveillance data is reported. A systematic approach to wildland fire respiratory health effects would be invaluable when attempting to address additional uncertainties from climatic change and population vulnerability.

**Figure 1:**
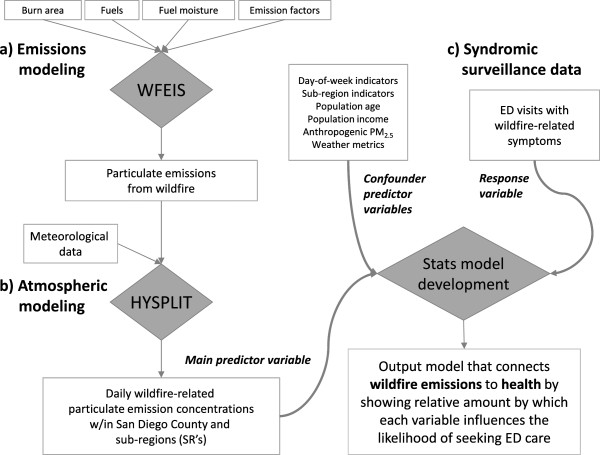
Schematic of modeling components showing: a) the fire emissions modeling (upper left), b) atmospheric transport modeling (lower left), and c) syndromic surveillance data set (upper right) which are compared to develop the predictive model of health outcomes due to wildfire particulate emissions (lower right).

The challenge of this multi-dimensional, interdisciplinary study was to develop an approach that is process-driven and built upon recent research in fire ecology, atmospheric modeling, and centralized health monitoring. Specifically, the overall challenge is the integration of various model and measurement-based components to properly understand the impact and predictability of wildland fire emissions on the human population. In our framework, there are three specific components to this integrated modeling activity: 1) modeling spatially-defined fire emissions taking into account vegetation fuels and weather, 2) accurately estimating particulate concentrations for downwind regions using transport/dispersion modeling, and 3) combining the smoke emission concentrations with electronic health outcomes monitoring to relate the modeled spatial/temporal distribution of pollution to the rates and spatial location of syndromic response outcomes (e.g. respiratory; Figure 1).

## Methods

**Figure 2:**
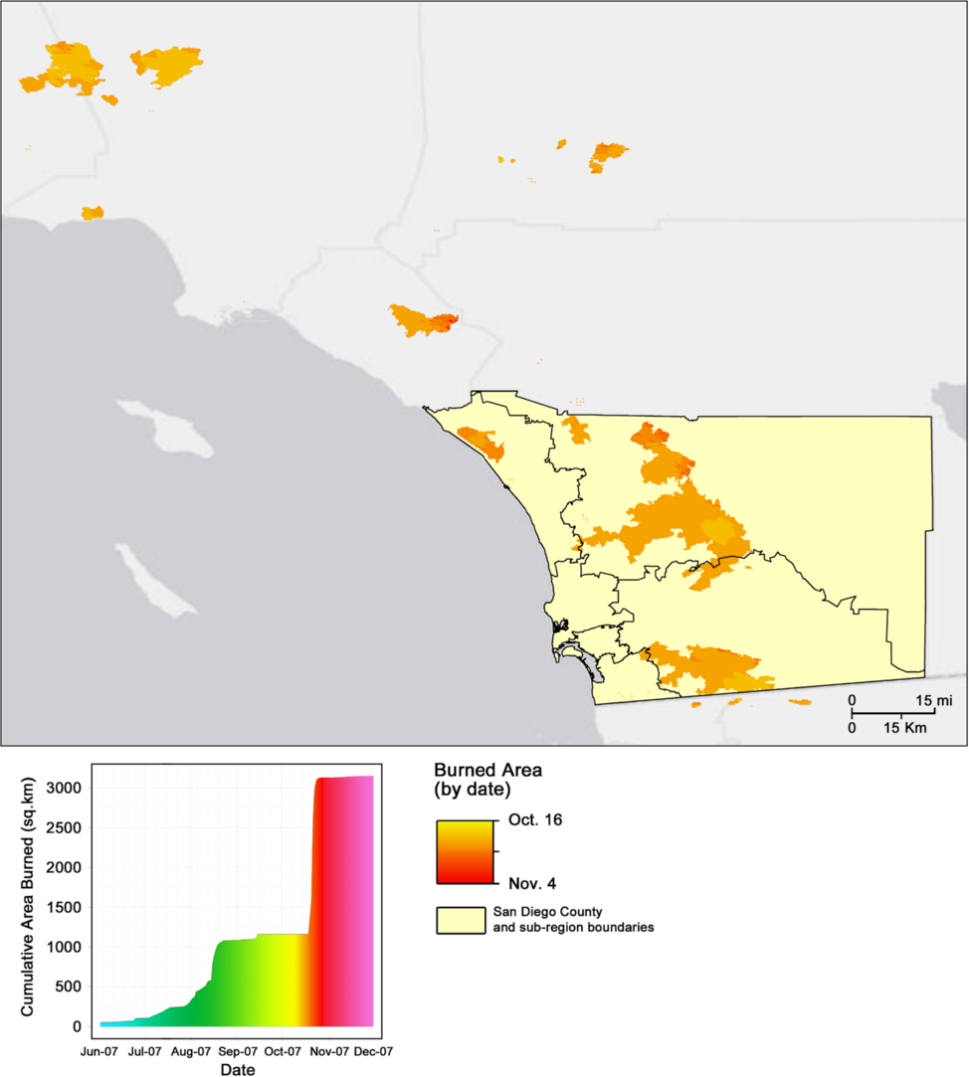
**Map of 2007 fires around San Diego County by date, and (bottom) cumulative burned area for the region surrounding San Diego County from June 2007 through December 2007.** Note the steep increase caused by the late October burn events (orange colors).

The region covered in this study is shown in Figure 2a along with a graph showing the cumulative area burned that year near San Diego County as a function of time (Figure 2b); the August and October burn spikes are clearly visible. The methods used to develop the integrated physical and statistical modeling framework include several major data components and operations (Figure 1). To address the purpose of this research, we utilized a mathematical exposure model to approximate the cumulative effects of duration and exposure to elevated smoke concentrations on respiratory symptoms. We then used a Generalized Additive Modeling (GAM) approach to use emissions-transport (via the mathematical exposure term), demographics, weather, and scheduling (day of week) information to derive an accurate predictive model that described the relationship between fire occurrence and respiratory-symptom-driven hospital visits; ED visits from the syndromic surveillance database serve as a proxy for determining the impact of fire on respiratory health in San Diego County.

### Fire emissions and atmospheric transport and dispersion modeling

The spatial maps of daily wildland fire-originating PM plume concentrations across San Diego County from January 1, 2007 through December 31, 2007 were developed by connecting a smoke transport model to a fire emissions model. The modeling system used spatial estimates of wildland fire emission (kg PM/m^2^) computed in the Wildland Fire Emissions Information System (WFEIS; Figure 1a) for specific times across Southern California as inputs to the transport model. Fires that influences air quality in San Diego County as determined using a back trajectory method were included in the model (fires shown in Figure 2a).

The WFEIS system is a geospatial tool designed to calculate spatially and temporarily resolved emission profiles for user-defined wildland fire burn geometries occurring in the conterminous United States and Alaska using the Consume fire emissions model [16]. French and others [17] provide an overview of the modeling methods and tool design in the supplementary material of the 2011 paper; details of the system and its operation are available on the system website [18]. One of the most valuable aspects of WFEIS is that it employs the Consume model in conjuction with mapped vegetation fuels, which are critically important in defining the amount and types of emissions within the smoke. Vegetation fuels are mapped based on the Fuel Characteristic Classification System (FCCS), developed by the US Forest Service to assess fuel characteristics that determine potential fire behavior, fire effects, and smoke emissions [19]. The fuels map used was obtained from the Landfire database [20] as a 30-meter resolution map for the region of interest. Fuel diversity is low for the burned area surrounding San Diego County, composed almost exclusively of shrubland chaparral. Similarly, WFEIS provides methods to use temporally relevant weather to drive the emissions calculations. Weather data from nearby Remote Automatic Weather Stations (RAWS [21]) was used to determine daily fuel moisture. Day of burning was determined from mapped fire perimeters with modeled fire progression using observed active fire detections from the MODIS active fire product [22]. This methodology provides spatially explicit information about fire progression and residual burning within known burn scars mapped at a daily time step as reviewed by Loboda and others [23]. The algorithm was applied within areas identified as “burned” within fire perimeters mapped by the US Geological Survey Monitoring Trends in Burn Severity (MTBS [24]).

**Figure 3:**
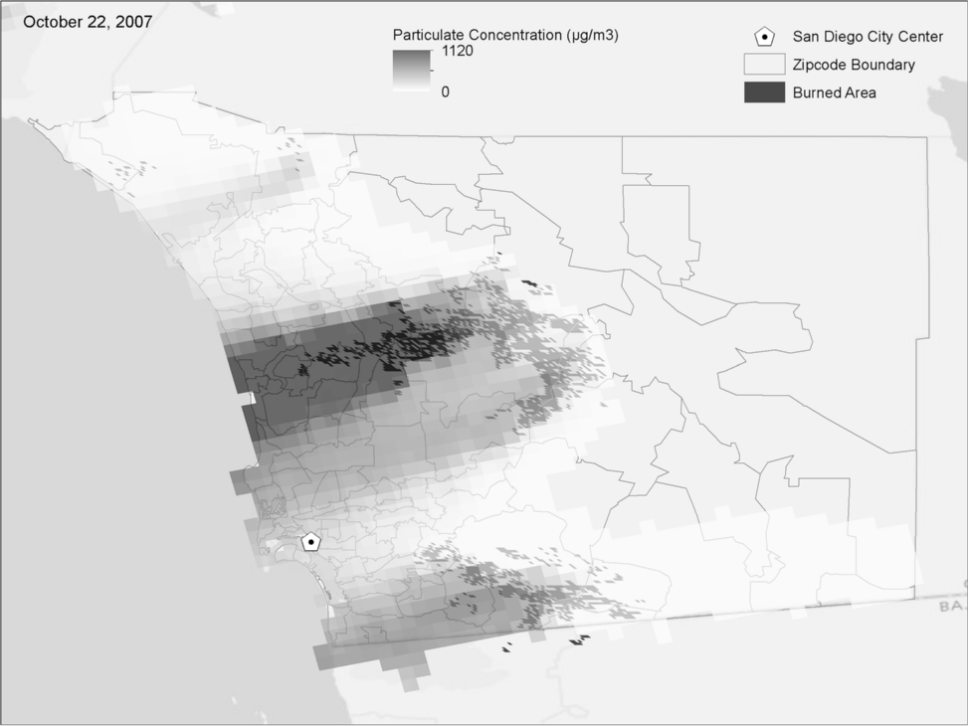
**Modeled smoke plume particulate (PM**_**10**_**) concentration on October 22, 2007, the peak day of burning.**

For this study, two independent and customized runs of the WFEIS software were used to generate daily PM_2.5_ and PM_10_ fire emission estimates using fire location and progression information derived from the modeled fire progression maps. These emissions are introduced into the Hybrid Single-Particle Lagrangian Integrated Trajectories (HYSPLIT) atmospheric transport model [25-27] to yield daily, one kilometer PM_2.5_ and PM_10_ concentrations (Figure 1b). The HYSPLIT runs use meteorological data from the National Weather Service’s National Center for Environmental Prediction (NCEP) Eta Data Assimilation System (EDAS [28]) and was run in its puff mode, which assigns a mass to each particle based on the mass emitted from the fire in the source cell and computes concentrations in downwind receptor cells. Fire emissions were converted into a 0.05 degree grid (approximately 5 km^2^) for input to HYSPLIT and released on an hourly basis. Once emitted, fire emissions were carried in the model for three days and concentrations were computed on a 0.01 degree grid (approximately 1 km^2^) on an hourly basis (an example is shown in Figure 3).

### Statistical modeling input data

**Figure 4:**
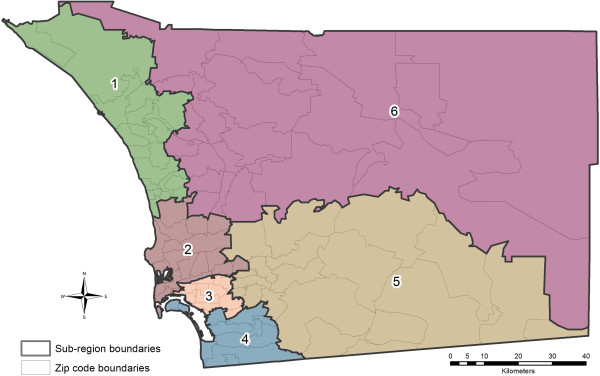
**San Diego County study area showing zip code boundaries in light grey with the modeled sub regions (SRs) outlined in dark black.** The analysis was performed at two geographic levels, the county region and the six SRs shown. The City of San Diego is in SR 3.

Input datasets to the respiratory effects model fall into four categories, which are described in this section: (1) atmospheric particulates, (2) syndromic surveillance health data records, (3) environmental conditions, and (4) demographic stratification. All datasets are spatially referenced to polygons defined by zip code. Two levels of geographic aggregation were considered; the first aggregation combines the full set of zip codes in the San Diego County study area (county region) and the second merges zip codes into sub-regions (SRs; Figure 4). The latter aggregation corresponds to six sub-regions that group adjacent zip codes according to a grouping method commonly used by County of San Diego Department of Health & Human Services Agency for official planning and evaluation purposes.

#### Atmospheric particulates

**Figure 5:**
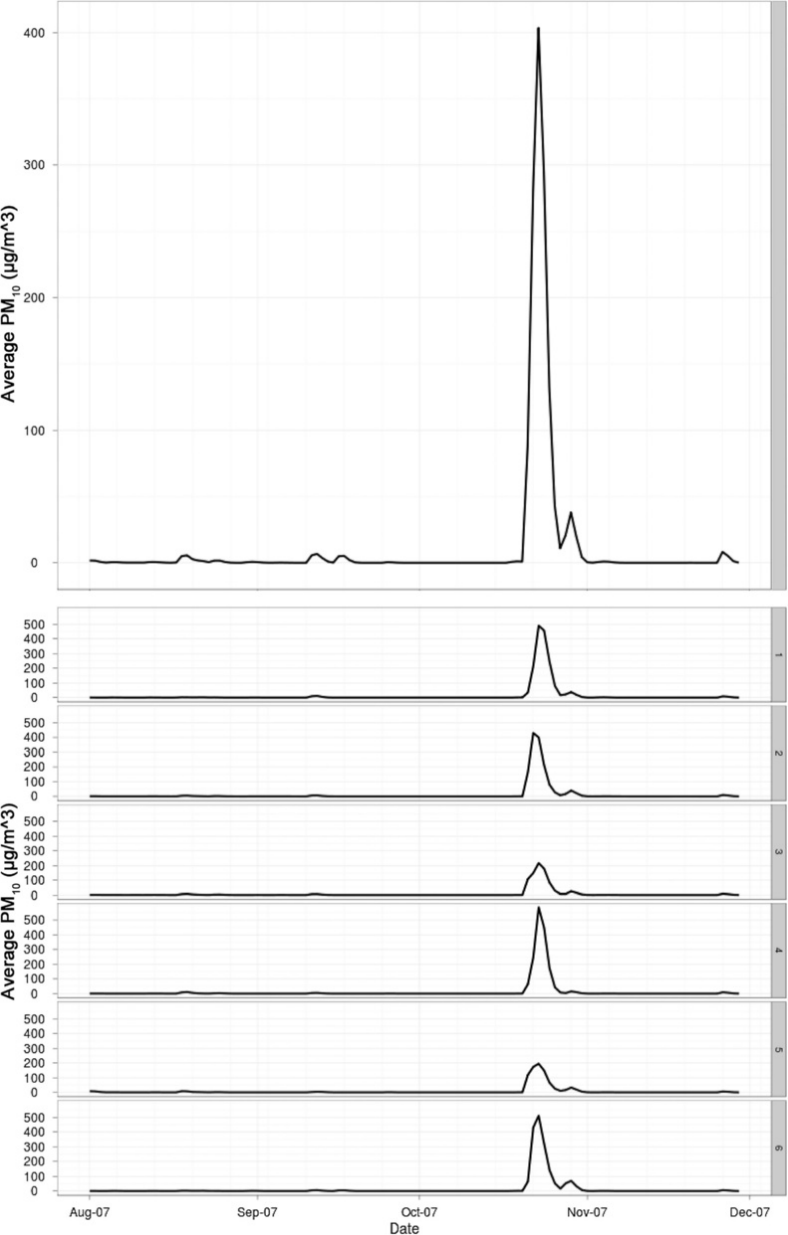
**Average fire-emitted particulates (PM**_**10**_**) for the San Diego County region study area (top), and for each sub region (SR; bottom).**

**Figure 6:**
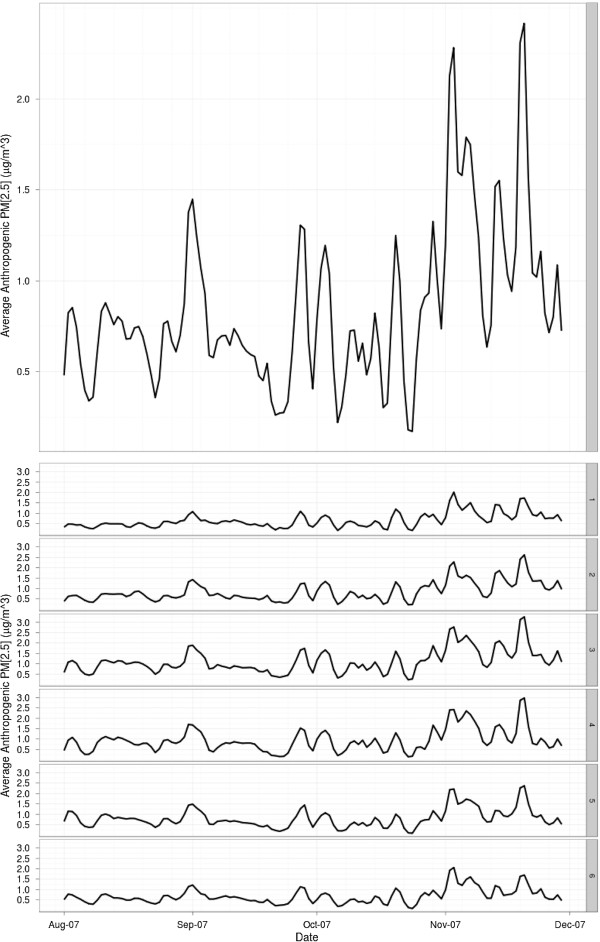
**Average modeled anthropogenic PM**_**2.5 **_**for San Diego County (top), and each sub region (SR; bottom).** (Note difference in Y-axis scale compared to Figure 5).

The atmospheric particulate inputs for the statistical modeling derive from two sources; the wildfire-emitted particulates derived from the emissions/transport model (average concentration over time are shown in Figure 5) described in the previous section, and the modeled anthropogenic PM_2.5_. Daily anthropogenic PM_2.5_ concentration surfaces were modeled with HYSPLIT based on the annual EPA NEI inventory 1999 base year. These daily modeled estimates were created at an equivalent resolution to the wildfire-emitted particulates and modeled using the same environmental inputs (average modeled anthropogenic PM_2.5_ is shown in Figure 6). Variations of HYSPLIT’s plume dispersion were tested against measurements of anthopogenic PM from air quality networks in San Diego County to optimize simulation time and model performance, resulting in the selection of the Gaussian-horizontal and Top-Hat vertical puff concentration distribution parameterization. HYSPLIT outputs were put into a spatial database coded according to their origin (i.e. fire vs. anthro) and time-stamp.

Concurvity (the analog of colinearity in linear models; [29]) exists between the wildland fire-emitted particulate matter classes of PM_2.5_ and PM_10_ resulting from the relatively short transport distances inhibiting mass differentiation between particle diameter classes. Paticulate matter classes are defined as all particulates smaller than the class designation. PM_2.5_ represents all particulates smaller than 2.5 μm, and PM_10_ represents all particulate smaller than 10 μm (including PM_2.5_). Particulate matter diameter classes have been found to have differential morbidity impacts [30] yet in our case the very close similarity in their concentration profiles makes them statistically indistinguishable. Therefore, the two classes of PM_2.5_ and PM_10_ were represented in the model as a single particulate matter variable (fire PM_10_) for fire-emitted particulates that ensures the modeled particle mass is unambiguously accounted for in the statistical model.

To account for the cumulative health effects of smoke on medical condition and address potential lag in seeking emergency care from smoke exposure, a simple truncated half-Gaussian cumulative-lag exposure weighting kernel was introduced on the fire PM_10_ variable. The kernel’s maximum weight was centered on the estimand day (location of 0) with a standard deviation of 1 day (scale of 1), weighting the current and previous days accordingly.

#### Syndromic surveillance heath data

Syndromic surveillance data from 2007 were obtained for the study from the County of San Diego Department Health and Human Services Agency. Syndromic surveillance data are de-identified and sent daily through secure mechanisms to the county health department. The syndromic surveillance data sources within SDADIC includes: hospital emergency department (ED) chief complaints, pre-hospital paramedic chief complaints, 911 call chief complaints, and medical examiner data. For this study the data used was hospital ED chief complaints.

The ED syndromic data are processed to identify key words or phrases among chief complaint data. These are then grouped into syndrome categories, which are then analyzed. In the process of grouping into categories, all chief complaints took into account accompanying information and used a generalized logic in whether to assign to a syndrome category or not [31]. For example, a chief complaint with key words such as, “cough with fever” would be categorized into the following core syndromes, “Fever”, “Influenza-like-Illness”, and “Respiratory”. Many encounters with mention of “Chest Pain” were not grouped into a study “chest pain” category due to accompanying information that linked this to an injury, abdominal pain, or post surgery. Free text parsing and processing is widely used among practioners in syndromic surveillance and numerous branches of effort have continued to further study and refine approaches for achieving an optimal level of sensitivity and specificity [32]. For this study, selected syndromes were identified to be included in the model for understanding wildfire effects on health and combined into a single variable. These study syndromes included asthma, respiratory problems, chest pain, and COPD.

**Figure 7:**
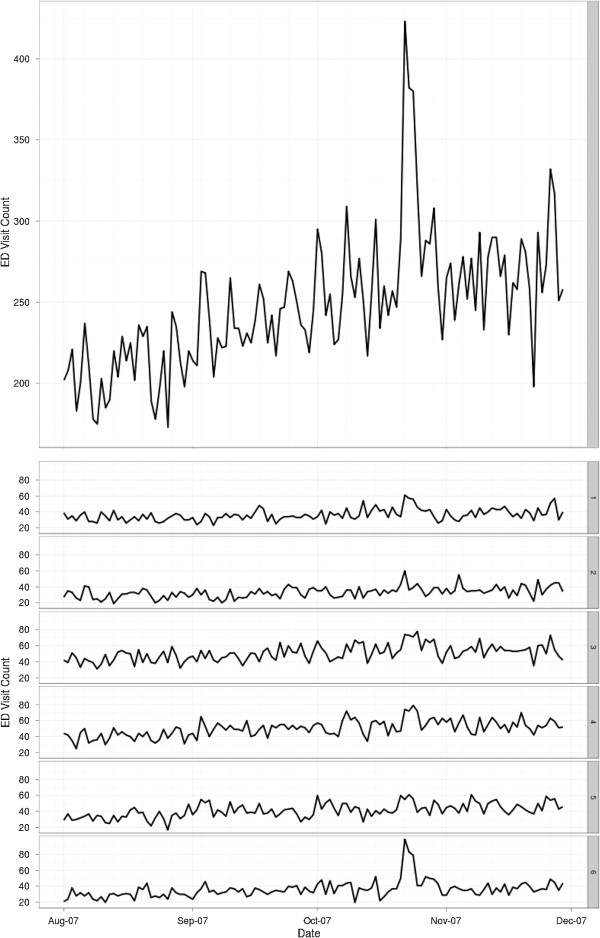
Daily ED visit counts for reporting hospitals in San Diego County (top) and by sub region (SR; bottom).

Data from SDADIC for this study were from 16 EDs from 2007 (Figure 7; Figure 1c). These data reflect approximately 86% of the total countywide ED encounters. Due to the unavailability of all hospital system’s data during the latter part of November and December 2007, analysis of the complete data set was limited to the time period of 08/01/2007 through 11/29/2007.

#### Additional model variables

It is known that relative humidity and temperature are significant respiratory irritants under correct conditions thereby effecting respiratory health [33]. Thus, in situ measurements of these variables were collected from a Remote Automated Weather Station database (RAWS [21]) and interpolated to zip code polygon centroids using a standard inverse-distance weighting scheme [34]. Population-weighted averaging was then used to aggregate the interpolated weather to the required model support geometry.

Demographic variables also are known to serve as factors in determining human health and human behavior of people to seeking medical care. Population data used in this study originated from a zip code dataset derived from the 2000 Census [35]. Based on heuristics and motivation for reducing the number of covariates, age groups were split into children/teenager and adult/elderly categories. In a similar fashion, two income categories were specified, attempting to delineate lower and middle-to-upper class households. Part of the motivation for these classifications is the assumption that there are behavioral differences, depending on age and income, in response to acute illness and how those individuals seek care normally and during an emergency situation [10]. Although they may be consequential, the interactions of these variables were not included in the modeling for this analysis. All demographic variables were included as proportions to normalize across the six SRs.

### Statistical framework

We used a generalized additive model (GAM) with binomial logistic regression [36] for relating the covariates to the health outcomes; more specifically, we modeled ED visit data from any day *i* and region *j*, as being binomial with probability *p*_*ij*_ and population size *n*_*ij*_ (known from demographic data) related to the covariates via the logistic link function:

(1) $$\mathrm{logit}\left(P_{\mathrm{ij}}\right)=\log\left(\frac{P_{\mathrm{ij}}}{1-P_{\mathrm{ij}}}\right)=\beta_{0}+\sum_{k=1}^{K}\beta_{k}x_{\mathrm{ij}}+\sum_{m=1}^{M}f_{m}\left(x_{\mathrm{ij}}\right)$$

where the first term on the righthand side (*β*_0_) is the offset term, the second term represents the linear portion of the model with coefficients that must be estimated, and the last term represents nonlinear functions, {*f*_*m*_}, which must be estimated. For the latter terms, we utilized a smoothing splines regression model which uses natural cubic splines and chooses the smoothing parameter via generalized cross validation [36]. To choose the appropriate smoothness of this spline model and minimize the effects of overfitting the data, we used a standard regularization method where the final smoothing parameter was selected via the so-called generalized cross-validation metric. It is critical to allow for nonlinearity within this model, since it is clear that the effects may not be linear over the range of inputs we expect to encounter. This modeling framework is quite flexible providing considerable capacity for modeling inputs in a realistic nonlinear fashion. Background on this model can be found in Wood [36]. In Table 1, we show a complete list of variables considered in this logistic regression. We considered interaction terms between selected covariates, and all continuous data inputs were tested for nonlinearity. Anthropogenic PM_2.5_ and the weather variables were determined to exhibit nonlinear characteristics thereby requiring nonlinear modeling.

**Table 1:** Terms tested for model development

**Model term**	**Units**	**County region**	**Sub region**	**Nonlinear**
		**Model**	**Model**	
**Particulates of Wildfire Origin**	**μg/m**^**3**^	**True**	**True**	**False**
**Anthropogenic PM2.5**	**μg/m**^**3**^	**True**	**True**	**True**
**Mean Daily Relative Humidity**	**Percent**	**True**	**True**	True*
Min/Max Daily Relative Humidity	Percent	False	False	NA
**Minimum Daily Temperature**	**F**	**True**	**True**	True*
Mean/Max Daily Temperature	F	False	False	NA
**Proportion Income > $50 k**	**NA**	**False**	**True**	**False**
**Proportion Age <24**	**NA**	**False**	**True**	**False**
**Is Monday**	**NA**	**True**	**True**	**NA**
**Is Tuesday**	**NA**	**True**	**True**	**NA**
Is Wednesday	NA	False	False	NA
Is Thursday	NA	False	False	NA
Is Friday	NA	False	False	NA
Is Saturday	NA	False	False	NA
Is Sunday	NA	False	False	NA
Is SR1	NA	False	False	NA
Is SR2	NA	False	False	NA
**Is SR3**	**NA**	**False**	**True**	**NA**
Is SR4	NA	False	False	NA
Is SR5	NA	False	False	NA
**Is SR6**	**NA**	**False**	**True**	**NA**
Housing/Population Density	NA	False	False	NA
Mean Elevation	meters	False	False	NA

Included terms, leading to the final model formulations for San Diego County, are indicated by ‘True’.

*Relative Humidity and Temperature combined as a bivariate nonlinear function.

Descriptive statistics for constant values and continuous input variables are reported in Table 2. Summaries are shown grouped by the San Diego County region and for each SR. Demographic variables included in the sub-regional models were normalized to proportions for each SR and remain fixed across all modeled response days as daily changes in demography are unavailable for the study area. These values are reported in Table 2a and continuous data inputs in Table 2b. Four model indicator variables that were found to be significant during testing (see Table 1) are also included: “Is Monday”, “Is Tuesday”, “Is SR3” and “Is SR6”. These indicator variables are defined as taking on the value of one when the variable is true and the value of zero otherwise.

**Table 2:** (a) Descriptive statistics for constant values and (b) continuous data inputs used in the SR model

**Observational Data/Model Term**	**San Diego County**	**SR 1**	**SR 2**	**SR 3**	**SR 4**	**SR 5**	**SR 6**
**a. Constant Values for subregional area model**							
*Proportion Age <24*	NA	0.37	0.32	0.40	0.41	0.36	0.36
*Proportion Income > $50 k*	NA	0.53	0.54	0.30	0.41	0.46	0.55
**b. Descriptive statistics (N = 121 days) for continuous data inputs**							
*Unique ED Visits*							
Mean	247.4	36.3	33.0	50.6	50.1	41.2	36.2
SD	40.8	7.3	7.0	9.9	10.2	8.9	10.9
Median	244	35	33	51	50	40	35
Range	173–423	23–61	19–60	31–78	25–79	17–61	20–99
*Particulates of Wildfire Origin (μg/m*^*3*^*)*							
Mean	11.73	15.56	12.99	8.16	15.19	7.28	14.67
SD	53.36	70.66	60.35	32.59	75.37	29.57	68.47
Median	0.14	0.11	0.11	0.09	0.10	0.08	0.10
Range	0–403.47	0–489.95	0–430.20	0–216.76	0–587.08	0–194.65	0–511.59
*Anthropogenic PM2.5 (μg/m*^*3*^*)*							
Mean	0.84	0.66	0.86	1.13	0.94	0.81	0.69
SD	0.44	0.36	0.47	0.58	0.55	0.44	0.36
Median	0.74	0.56	0.73	1.03	0.84	0.69	0.60
Range	0.17–2.42	0.17–2.01	0.22–2.62	0.23–3.26	0.14–2.98	0.09–2.37	0.09–2.06
*Mean Daily Relative Humidity (%)*							
Mean	39.66	35.38	42.27	43.68	42.37	39.49	35.44
SD	14.87	14.05	15.92	15.85	15.13	14.75	15.13
Median	42.77	35.14	45.39	47.46	46.77	42.86	36.85
Range	6.07–78.65	6.06–77.95	5.85–80.07	6.01–78.57	5.89–74.46	6.11–76.56	6.47–83.68
*Minimum Daily Temperature (F)*							
Mean	54.34	50.99	52.56	56.75	56.85	55.70	54.18
SD	7.75	8.78	6.90	7.38	7.48	7.60	8.88
Median	54.14	51.21	51.89	56.53	56.77	55.26	54.20
Range	36.48–71.73	25.48–69.58	36.36–68.52	38.42–73.42	34.61–73.27	39.70–72.99	31.60–73.60

## Analysis and results

### Variable and model selection

An important aspect of the final model is whether dependence is linear or nonlinear and also if the dependence is independent additive or involved in a bivariate interaction with another variable. A standard method for determining whether a model term should be nonlinear (versus including the term as a linear predictor) focuses on assessing the fitted spline function and associated confidence bands to determine if confidence bands (on the function) may accommodate its linear counterpart, i.e. a straight line could be drawn within the confidence intervals [33]. We are also looking for parsimony in our model, and this simple approach is insufficient to justify including a nonlinear term if no fundamental ancillary evidence for a nonlinear relationship with the dependent variable exists or can be substantiated. Hence, interpretation of models, including nonlinear terms, must be approached with care and re-interpreted as model terms cycle in or out; standard model term selection metrics can be misleading without visual assessment and confirmation.

All continuous variables were repeatedly tested for nonlinearity through the “plot and confidence bound method.” Static predictors and continuous terms identified as linear predictors were selected via a backward variable elimination procedure – for this, a threshold significance value of 0.05 was nominally used, although most included terms generally had significance values far below this level. Significance of the nonlinear model terms was assessed through a combination of approximate chi-square p-values and confidence bands on the functional forms. Table 1 contains a list of all variables tested and selected during the variable selection phase.

**Figure 8:**
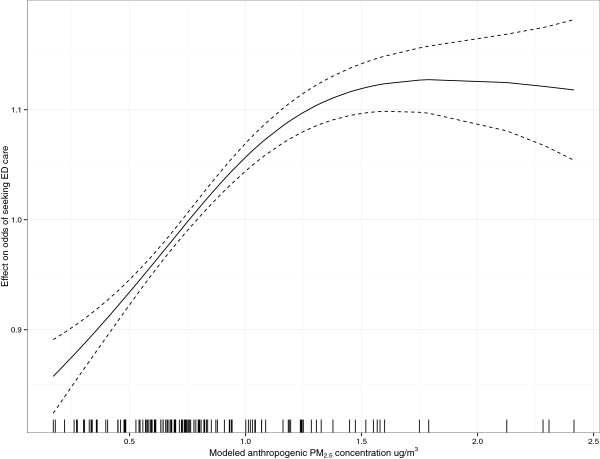
**Rugplot for anthropogenic PM**_**2.5 **_**nonlinear model terms for San Diego County region showing the spline fit as solid black line and confidence intervals as dotted lines.** The rugplot representing the model for the SRs is similarly shaped.

An example of the modeling results to assess effects of anthropogenic PM_2.5_ in the San Diego region on ED visits is shown in the rugplot presented in Figure 8. The assumption that this is truly a nonlinear relationship is derived by considerations of the estimated confidence bands on the function. The shape of the rugplot indicates that the relationship is linear at lower PM_2.5_ concentration levels but then drops and becomes curvilinear as anthropogenic PM_2.5_ moves to higher levels. Therefore, a nonlinear relationship is confirmed based on the fact that any predictive linear function drawn from the origin, over the range of the predictor variable data, would extend outside of the confidence bands on the spline.

Some previous long-term synoptic air quality studies investigating nonlinearities and threshold effects between air quality and mortality have found no justification for nonlinear functional forms for air quality predictors [33]. It is possible that the anthropogenic PM_2.5_ spline (Figure 8) may approach a linear form with additional data points, especially at higher concentrations, but given the uncertainty at higher concentrations we felt the threshold at these high concentrations would not unnecessarily enhance the high concentration effect. Fire-emitted PM_10_, on the other hand, demonstrated strong linear behavior with increasing concentrations.

**Figure 9:**
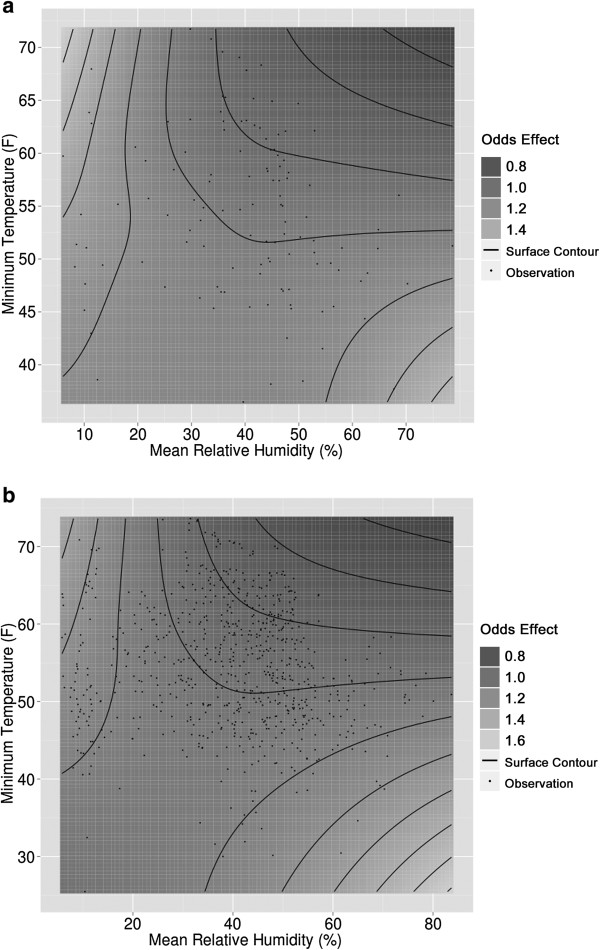
**Contour representation of the humidity/temperature bivariate function for odds effect. (a)** Bivariate rugplot for the weather function odds effect for the San Diego County region model. **(b)** Bivariate rugplot for the weather function odds effect for the SR model.

We expected interactions between relative humidity and temperature as well as threshold effects at the most extreme observed weather values. We fit a bivariate function using a tensor product spline model based on univariate natural cubic splines, which is recommended for fitting bivariate splines of two variables with differing measurement units [36]. Contour representations of the bivariate functions for the county region-level and SR-level models are shown in Figures 9a and b. The contour plots reveal an inverse relationship between minimum temperature and mean relative humidity in terms of odds effect; an increase in one variable associated with a decrease in the other variable causes the odds to rise. The highest odds are observed at moderate (minimum) temperatures and higher humidity, possibly evening or nightime. When both parameters approach their maximum together, odds decrease. One explanation is that combined high humidity and high temperature results in reduced activity level and, hence, less exposure during particularly humid, hot periods. The function estimates between the models, by qualitative observation, appear stable because they generate similar patterns of interaction between relative humidity and temperature. The contour plots indicate a nonlinear model is appropriate for including temperature (as maximum daily temperature) and relative humidity (as mean daily relative humidity) in the predictive models.

### Model diagnostics and results

**Figure 10:**
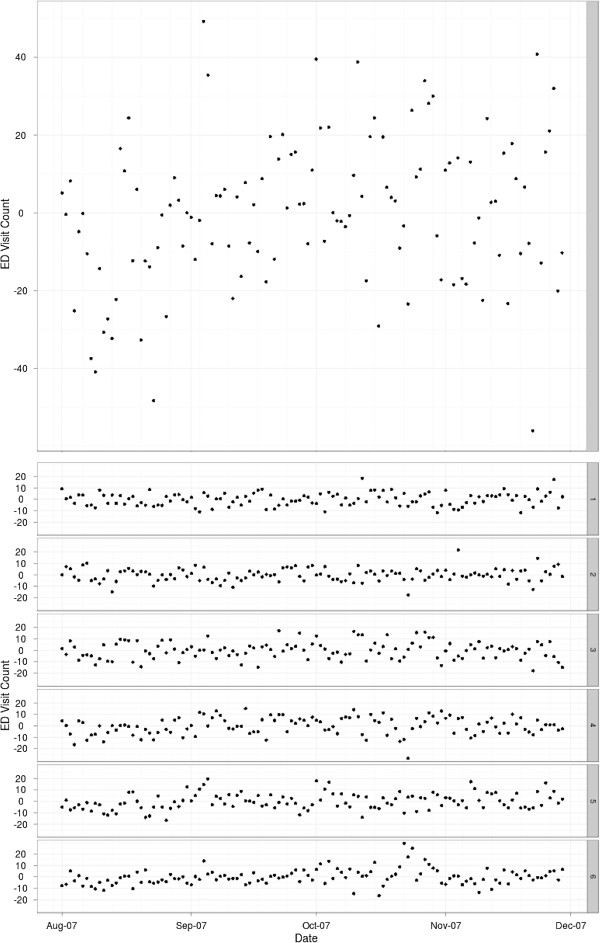
Unstandardized model residuals for (top) San Diego County region and (bottom) the six SRs.

Summaries of the overall best fit models for the San Diego County region and SR models are presented in this section. The deviance residual plots for these best fit models are given in Figure 10. These plots are useful in looking for patterns suggesting significant violations of the underlying Generalized Additive Model assumptions, and in particular we are looking for temporal correlation between residuals, significant outliers, and temporally changing patterns in the variance. A review of the deviance residual plots for these best-fit models (not shown) indicate that model residuals for both aggregation levels generally appear normally distributed with some exceptions occurring in early August for some SRs where a pattern of over-estimation occurs before returning to normality a few days later. Also, a degree of temporal correlation is present in the SR model residuals not visible in the aggregated dataset. Incorporating a two- or three-day moving average would likely collapse these correlations, but such an approach would mask the weekday and SR indicator effects that figure prominently in the final models. Hence, there may be a degree of overdispersion in the SR model leading to lower variance estimates on coefficient values due to residual correlation. The residual correlation observed in the SR model hints that there are additional model terms not included. In general, the model diagnostics for the SRs suggests there may be some minor modifications that would improve the model fit, but taken in total, these diagnostics confirm that the statistical assumptions for the selected model are reasonably satisfied.

**Figure 11:**
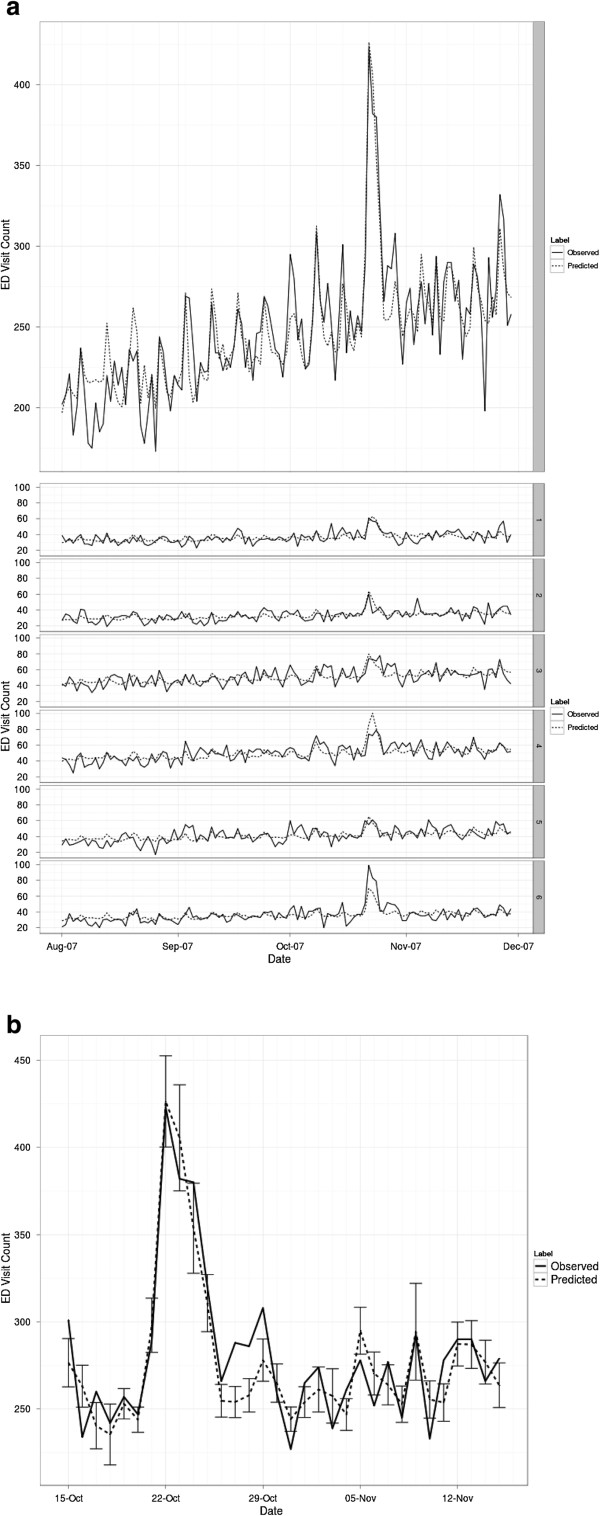
**Plots of observed and estimated ED visit counts. (a)** Observed ED visit counts plotted against model estimates for the full time period modeled for the San Diego County region and the six SRs. **(b)** Observed ED visit counts plotted against model estimates for the times directly before, during, and following the October 2007 fire event with standard errors for the estimates shown as error bars.

A time series of estimated and observed ED visits for the San Diego County-level model is shown in Figure 11a and a subset of the graph for the times directly before, during, and following the October 2007 fire event are shown in Figure 11b with error bars. The model performs well (deviance explained is 76.6%; Table 3) capturing the increasing seasonal trend in ED respiratory-related visits and inter-day variability related to the weekend effect. Two exceptions are mid-August and the PM_10_ spike following the October burn event. The August deviation is likely a result of this being a high-vacation period, while the late October spike deviation is difficult to attribute. ED visits are under-estimated by roughly 15% for these days.

**Table 3:** **Model results for the logit regression (Equation** 1**)**

**Model results summary**										
	**San Diego County**					**Sub region**				
**Term**	**R**^**2**^** (adj)**	**Deviance explained**	**Estimate**	**Standard error**	***p***	**R-sq. (adj)**	**Deviance explained**	**Estimate**	**Standard error**	***p***
Intercept			−7.87E-5	5.75E-07	<0.001			4.26E-4	1.27E-4	<0.001
Particulates of Wildfire Origin (μg/m^3^)			1.0010	.0009	<0.001			1.00093	.00012	<0.001
Is Monday			1.172	.020	<0.001			1.174	.019	<0.001
Is Tuesday			1.075	.019	<0.001			1.072	.018	<0.001
Is SRA3	**0.75**	**76.60%**	NA	NA	NA	**0.63**	**75.80%**	.512	.018	<0.001
Is SRA6			NA	NA	NA			1.209	.029	<0.001
Proportion Income > $50 k			NA	NA	NA			.0087	.0024	<0.001
Proportion Age <24			NA	NA	NA			5.584	2.915	<0.001
F(Anthropogenic PM2.5)			NA	NA	<0.001^*^			NA	NA	<0.001^*^
F(Relative Humidity, Temperature)			NA	NA	<0.001^*^			NA	NA	<0.001^*^

Coefficients indicate the effect on modeled odds of the observation values.

*Chi-squared test.

Results for the SR-level model (Figure 11a) show there is a marked under–estimation in SR6 during the peak fire period. As shown in Figure 4, this geographic region is the largest in San Diego County and because of this, there is considerable heterogeneity in the particulate matter concentrations across its member zip codes. However, each zip code in the SR captured modeled particulate matter at some point during the plume circulation so there is not a justification for any zipcode to be removed. Based on preliminary results, we decided to use a zipcode population-weighting approach for spatial aggregation and this will alleviate some of these impacts, but ultimately a low estimation pattern persists in SR6. We conjecture that removing low PM_10_ concentration zipcodes would likely further improve the estimation but the desire for completeness in the syndromic coverage area resulted in their inclusion.

Table 3 reports the estimated coefficients for the logistic regression model, presented in Equation (1). The effects of these coefficients relative to their impact on the likelihood for seeking emergency care are shown in Table 4. Specifically, to understand the impact of coefficients, one needs to assess the original logistic regression coefficient (from Table 3) in combination with the range of the predictor variable. For example, we compute the effective range of the total predictor contribution within the logistic regression model, and then we can compute the total range of that predictor variable relative to additive effect for the odds of seeking emergency care. Table 4 shows these ranges in odds along with the ranges of the original predictor variables for both the regional and sub-regional model. Note that the indicator variables included as offsets (i.e., day of week, SR designation) introduce a static change in odds if the variable is positive while the linear and functional variables (i.e., fire PM_10_, population age) odds effects change with the predictor value. For example, in Table 4, the transformed coefficient for the Monday indicator variables is 1 on non-Mondays and 1.17 on Mondays. This can be interpreted as follows: on Mondays, the odds that a person seeks ED care increases by 17% for both the regional and sub-regional model. On Tuesdays, the effect is less pronounced with a 7% increase in the odds of seeking ED care for both models. An example of a variable with continuous data is that of the fire-emitted PM_10_ (first line), where the original logistic regression coefficient from Table 3 may at first appear small, but when scaled to account for the peak mean modeled concentrations, the resulting odds increase is approximately 41% (regional) and 72% (sub-regional) as shown in Table 4. Hence, combining the model coefficients and the nonlinear regression function plots indicate an overwhelming influence of fire-emitted PM_10_ on the odds of seeking medical care at high PM concentrations.

**Table 4:** Transformed model coefficients showing the direct additive effect on odds given an observed variable’s dynamic range

**Odds effects summary**				
	**San Diego County**		**Sub regions**	
**Term**	**Observed data range**	**Estimated odds effect range**	**Observed data range**	**Estimated odds effect range**
Intercept	−9.45	7.87E-05	−7.76	4.26E-04
Particulates of Wildfire Origin (μg/m^3^)	0 – 403.47	1 – 1.41	0 – 587.08	1 – 1.72
Is Monday	{No, Yes}	Yes – 1.17	{No,Yes}	Yes – 1.17
Is Tuesday	{No, Yes}	Yes – 1.07	{No,Yes}	Yes – 1.07
Is SR 3	NA	NA	{No,Yes}	Yes – 0.51
Is SR 6	NA	NA	{No,Yes}	Yes – 1.20
Proportion Income > $50 k	NA	NA	0.30 – 0.55	0.24 – 0.07
Proportion Age <24	NA	NA	0.32 – 0.41	1.74 – 2.01

The values for ‘Estimated Odds Effect Range’ are based on GAM with binomial distribution and logit link.

The additional variables included in the sub-regional model show spatially specific effects on odds within SR3 (51% decrease in odds of seeking ED care) and SR6 (20% increase; Table 4). Of the six SRs, SR3 has the second highest proportion of population under the age of 24 and lowest proportion of income above $50,000 (Table 2b). The coefficients of these demographic variables in Table 4 show that an increase in proportion of wealthy households (>$50 k) leads to a decrease in ED visit odds while an increase in proportion of population below 24 years increases the odds. This suggests that access to health care may vary by geography and therefore lead to differing levels of health outcomes following a wildfire.

## Discussion

In this paper we review the coupling of a geospatial model of wildfire particulate emissions and transport within a GAM statistical modeling approach to create an empirical model of respiratory health outcomes under specific smoke transport scenarios. Another recent study has demonstrated a similar use of syndromic surveillance data in North Carolina [11] which emphasizes the important advances and opportunities taking place within electronic health data collection and the vegetation fire emissions community. While the North Carolina study [11] used similar health data, they used a very different method for mapping smoke concentration. The North Carolina study [11] as well as the earlier study from San Diego of the 2003 wildfire events [7] used satellite-based assessments of PM concentration based on sensing of Aerosol Optical Depth (AOD), while our study used modeled concentrations based on the location and dispersion of smoke from known fires affecting the region. Similarly, research on 2003 fires in British Columbia [14] used modeled smoke concentration based on fire location and PM dispersion, but with a different set of data and tools from our study. Both approaches to smoke concentration mapping have been shown to be reliable methods, but the two have not been rigorously compared, a task that would be of value for development of operational methods. As improvements in the physical modeling of smoke concentration mapping and forecasting by USFS, NOAA, EPA, and other efforts are made, development of methods to use these model results in conjunction with electronic health data sets are important to develop. The work presented in this article demonstrates a rigorous method to connect PM concentrations from wildfires to health outcomes that takes advantage of developments on the two fronts of geospatial fire emissions and smoke mapping, and electronic syndromic surveillance health information systems.

The previous studies cited employed somewhat different generalized linear regression modeling approaches. In particular, they used a Poisson repeated-measures linear regression [7], logistic linear regression with repeated measures [14], and Poisson linear regression with general cumulative risk estimation over lag days [11]. The present study utilizes a binomial logistic regression with a fixed lag function, which is very close to a Poisson regression for these large populations, but with the extension of allowing for linear and nonlinear spline functions. It was felt that the extension to allow for nonlinear dependency, especially related to PM_2.5_, humidity, and temperature, would be important and enable a more accurate estimation for our scenario.

The statistical modeling was carried out at two levels of spatial aggregation grouped by zipcode: (1) San Diego County and (2) sub-regions of San Diego County. The county-level analysis is easier to interpret having fewer model terms and serves as a heuristic, while the sub-regional approach better captures spatial variability and allows for the inclusion of additional spatially specific demographic variables. We believe that a finer regionalization focus with more spatially specific demographic variables, especially at the level of the zipcode, could prove more accurate and useful. In particular, a more fine-grained model would be particularly attractive from the viewpoint of predicting specifics of health outcomes linked to environmental events. However, it is also the case that when approaching a finer spatial scale model more underlying variations cannot be accounted for by the explanatory variables and/or the model. A spatial coarsening helps to smooth out these effects via averaging. Hence, it would be an interesting study to determine what spatial scale is best in terms of balancing the effects of data resolution on model performance.

An important aspect of the model is that by virtue of having a statistical model (GAM logistic regression) there is the ability to provide uncertainty bands on estimations. We have not shown any specific results along these lines in this paper, but the framework readily supports that these bands may be useful from the viewpoint of bounding the estimate, and quantifying worst-case scenarios.

In terms of the future evolution of the framework, it is not necessarily expected that the exact model form here will persist if our approach is merged with additional syndromic studies. In particular, it is likely that the regression functions for some variables may change from linear to nonlinear or vice versa, but the overall approach for determining this would be the same. In addition, the focus of this study is ED encounters and is not intended to capture long-term or latent health effects for the general population related to wildland fire particulates—justifiably so within the constraints of the dataset’s spatiotemporal characteristics (residence zip code of cases). But it is quite straightforward to incorporate more inputs or utilize a different or expanded variation on the health-effects variables.

An additional note is that since we were working with ED data, the modeling results realistically support only general modeling conclusions for the population seeking acute care, and further work and expertise is needed before assuming these conclusions carry over into the general population. As an example, there is some argument based on studies of respiratory impacts from events such as wildfire smoke exposure to assume that the PM impacts are linear. However, as discussed above and shown in Figure 8 we found that the available ED data supported modeling the impacts of anthropogenic PM as a non-linear function, with a threshold value after which the effect on the ED visits seems to flatten out. While a full evaluation of the influence of anthropogenic background PM would be valuable, our study was not in a position to do so since we did not employ more data than the year of the fires. In the future it may be of value to (a) utilize more data and investigate whether this threshold-effect is confirmed, and (b) if it is confirmed, more seriously evaluate/quantify these threshold values of PM concentration which the general population is exposed to which result in an increase in ED visits. If available, this type of information could be used to more accurately predict specific health behavior during a fire and thus improve disaster response to reduce or prevent a certain level of hospitalization.

The research presented demonstrates the development of predictive tools that could be of value for forecasting and planning for the future. An important aspect of the resulting model is its generality thus allowing its ready use for geospatial assessments of respiratory health impacts under possible future wildfire conditions in the San Diego region. Under a separate analysis for this project the authors assessed weather conditions conducive to fire using methods reviewed in Loboda and others [37]. Canadian Fire Weather Index was calculated for the study area using weather variables produced by the Regional Climate Model (RegCM v. 4.1 [38]) under Intergovernmental Panel on Climate Change (IPCC) future climate scenarios for 2001–2040. At the decadal scales the RegCM produced conditions comparable to those observed during 2001–2010. This result indicates that San Diego County will experience approximately two extreme fire seasons each decade by 2040, similar to the present. Based on the specific characteristics of those future fires (e.g., location, spatial/temporal evolution, and local weather conditions), one can use the developed models to understand and predict the impact on the respiratory health in the region; this is not just an overall effect (total number of people affected), but rather it is a prediction that provides specific joint temporal and spatial information on respiratory effects.

The coupled statistical and process-based modeling demonstrates an attractive end-to-end method for generating reasonable estimates of wildland fire particulate matter concentrations and health effects at resolutions compatible with resolutions (temporal and spatial) that syndromic surveillance data is reported. A beneficial feature of the approach is that the model is easily extensible, i.e., it can readily be updated and/or extended as more data (in time or additional variables) becomes available. Also, the model may be readily generalized to other regions and conditions, it may include/filter through a significant number of predictor variables (demographic and weekly/seasonal), and it could easily operate on finer or coarser spatial/temporal scales. As demonstrated in this paper, the method is adaptive in automatically being able to include both linear and nonlinear dependencies, after determining which is appropriate, and it can include interactive dependencies between predictive variables – an example of the latter was shown for the variables of humidity and temperature. Finally, the methods have a theoretical foundation that automatically supports rigorous computing of uncertainties, i.e., confidence bands on the model parameters, and prediction bands on the expected health outcomes. The latter could be very valuable information to healthcare professionals enabling them to have the ability to bound the scope of an impending health care issue.

As for areas for research and extension, an important consideration not addressed in the present framework is that the predictor variables themselves are reported with differing levels of error. This provides motivation for applying more general “errors-in-variable” methods within the GAM logistic regression – the results will be more accurate parameter estimation and prediction, along with statistical confidence intervals and significance values that are more statistically valid and hence reliable. This has been an active area of research within the statistics community, and there are number of attractive and theoretically supported approaches for addressing this problem [39]. Another important extension will be that of trying to incorporate more dependencies of the predictor variables as the need becomes more apparent. One alternative framework for this would be that of multivariate adaptive splines, combining a local principal components analysis with multivariate regression splines [40]. Improvements to the developed framework, whether statistical or related to spatial scaling or database development, will benefit the public health community by providing a quantitative understanding of how wildland fire can impact a community, and can provide guidance on how a community could prepare for wildland fires in the future.

## Conclusions

This paper presents a review of methods and results of a study to connect the impacts of wildfire particulate emissions to respiratory health outcomes, as measured using ED visits collected through the SDADIC syndromic surveillance system. The model performs quite well at estimating ED visits in most cases (Figure 11) by including nine ancillary variables in addition to wildfire smoke concentration. While model coefficients and functional estimates are specific to San Diego County, the method itself is general and has applicability to other regions and syndromic responses. Model results from this study for the 2007 season, show that at peak fire particulate concentrations the odds of a person seeking emergency care is increased by approximately 50% compared to non-fire conditions (41% for the generalized case, 72% for a geographically specific case; see Table 4). In addition demographic variables, specifically income level and age, were shown to also have some impact on these odds results. Finally, the standard regression diagnostics involving the residuals confirmed that the statistical regression model fit was satisfactory. The model has relevance for application for future climates, since it is linked to weather, and allows for an improved understanding of how change in climate in the San Diego Region could impact respiratory health.

## Abbreviations

PM: Particulate matter; SDADIC: San Diego aberration detection and incident characterization; COPD: Chronic obstructive pulmonary disease; GAM: Generalized additive modeling; WFEIS: Wildland fire emissions model; HYSPLIT: Hybrid single-particle lagrangian integrated trajectories; ED: Emergency Department; RAWS: Remote automated weather station database.

## Competing interests

The authors declare that they have no competing interests.

## Authors’ contributions

All authors worked as a team to discuss and develop the detailed tasks required to complete the research reported here. All authors were involved in writing and editing the manuscript. BT provided expert guidance on the design of the statistical modeling framework, application of the statistical model, and interpretation of the model results as well as conducting some data analysis. NF provided project coordination, guided the development of the fire emissions model, and provided expertise in running the emissions model. BK provided input to the statistical model development and implemented the concepts of the model in the statistical software, conducted the statistical tests, created the model outputs, and provided input on interpretation of the model results. MB ran the fire emissions model, assisted BT and BK in application of the statistical model concepts, and assisted RCO in executing the smoke concentration model. RCO implemented the HYSPLIT smoke transport model and created the smoke concentration estimates. JJ provided expertise in syndromic surveillance data and provided the health data sets used in the analysis. MG provided expertise in utilization of the health data sets and interpretation of model results. TL provided burn scar datasets and additional input on the fire characteristics that were used in the emissions model and data on future fire predictions that were used in interpreting the model results. SW provided expertise in utilization of the PM modeling and interpretation of model results. All authors read and approved the final manuscript.
